# Synthetic CT artifacts in MR‐only brain radiotherapy: Clinical implementation experience

**DOI:** 10.1002/acm2.70733

**Published:** 2026-08-03

**Authors:** Hyeri Lee, Ahmet S. Ayan, Sasha Beyer, Dukagjin Blakaj, Dominic DiCostanzo, John C. Grecula, Joshua Palmer, Raju Raval, Raj Singh, Evan Thomas, Michael Weldon, Xiangyu Yang, Wesley Zoller, Nilendu Gupta

**Affiliations:** ^1^ Department of Radiation Oncology The Ohio State University Columbus Ohio USA; ^2^ Department of Radiology The Ohio State University Columbus Ohio USA

**Keywords:** MR‐only workflow, quality assurance, synthetic CT

## Abstract

**Background:**

Magnetic resonance imaging (MRI) provides superior soft tissue contrast compared with CT and is essential for delineation of targets and organs at risk in brain radiotherapy. Because CT is traditionally required for dose calculation in radiation therapy, synthetic CT (sCT) generation from MRI has enabled the development of MR‐only workflows. Prior studies have primarily focused on dosimetric equivalence between CT‐ and sCT‐based treatment plans, with limited emphasis on practical implementation challenges encountered during clinical use.

**Purpose:**

This study aimed to share clinical experience implementing an MR‐only brain radiotherapy workflow, with a focus on practical challenges encountered.

**Methods:**

Twenty‐eight patients underwent a dedicated MR simulation protocol for sCT generation in addition to standard‐of‐care planning CT simulation. MRI acquisition was performed using an immobilization mask with a dedicated head coil positioned over the mask. The sCT protocol consisted of four sequences: T1 VIBE Dixon, T2 SPACE, PETRA, and TOF‐MRA. Clinical treatment plans generated using the planning CT (pCT) for photon therapy were recalculated on the corresponding sCT datasets.

**Results:**

Twenty‐eight patients were scanned, and 25 synthetic CT datasets were successfully generated. Nine sCT datasets required rigid re‐registration because of inter‐sequence patient motion. Findings were categorized into workflow‐related sCT challenges, including inter‐sequence motion, suboptimal coil placement, and external localization marker interference, and algorithm‐related imaging artifacts, including HU misclassification at tissue/bone/air interfaces and sCT degradation in post‐operative anatomy with surgical meshes.

**Conclusion:**

Clinical implementation of a brain MR‐only workflow identified two major categories of challenge: workflow‐related sCT perturbations and algorithm‐related imaging artifacts. This distinction has important implications for mitigation strategies. Case‐specific sCT review, structured eligibility screening, and prospective site commissioning are essential for safe clinical deployment. Practical commissioning and per‐patient QA checklists are provided as supplementary materials.

## 1 INTRODUCTION

In radiation oncology, CT imaging plays a critical role in treatment planning by providing geometrically accurate anatomical information and electron density data necessary for radiotherapy dose calculation. However, CT has relatively poor soft tissue contrast compared with MRI. Consequently, radiation oncologists frequently request additional MRI examinations to improve visualization of targets and organs at risk (OARs), particularly in anatomical regions such as the brain and pelvis.

Conventional CT simulation workflows, often followed by an additional MRI may introduce treatment delays and logistical complexity. The development of synthetic CT techniques has enabled MR‐only treatment planning workflows by deriving Hounsfield unit (HU) values directly from MRI datasets.

Early approaches relied on bulk density assignment methods in which uniform electron density values were assigned to segmented tissue classes such as bone, soft tissue, and air.^1^, ^2^, ^3^ More recent techniques employ atlas‐based approaches^4^, ^5^, ^6^, ^7^ or deep learning–based algorithms^8^, ^9^, ^10^ to assign HU values to MRI voxels. Current MR‐only literature primarily emphasizes dosimetric equivalence obtained under controlled study conditions, often without accounting for practical clinical variability and imaging imperfections encountered during implementation.

Therefore, evaluation of MR‐only workflows under realistic clinical conditions remains important. This report describes our institutional experience implementing an intracranial MR‐only workflow using an atlas‐based sCT algorithm, with a particular emphasis on identifying practical implementation challenges and quality assurance considerations rather than systematic dosimetric validation.

## 2 MATERIALS AND METHODS

**FIGURE 1:**
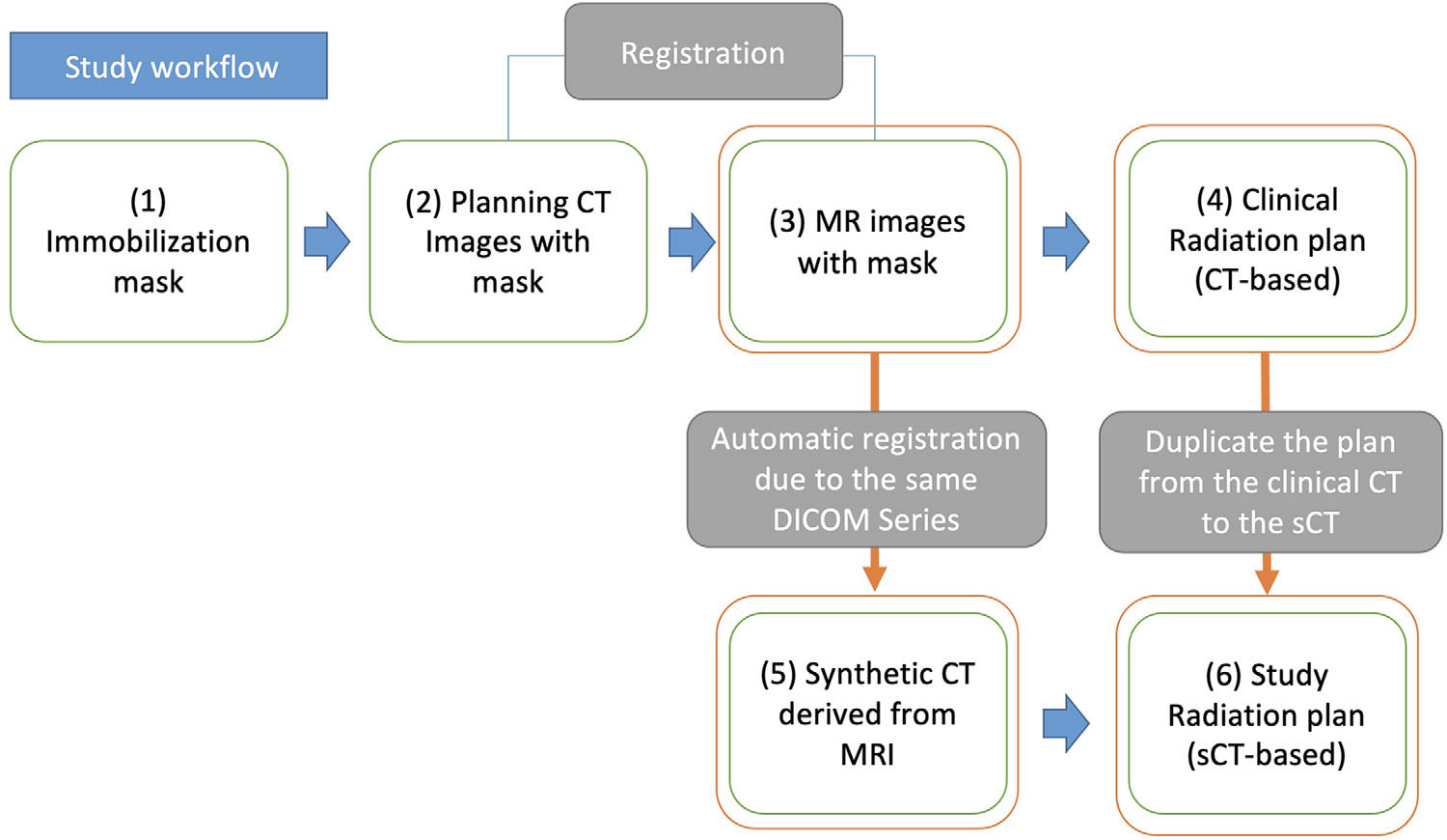
Scheme of the study workflow.

As part of clinical implementation, 28 patients were prospectively recruited for this study. A schematic overview of the workflow is shown in Figure 1. All patients first underwent standard‐of‐care planning CT (pCT) simulation. A custom immobilization mask fabricated during CT simulation was subsequently used during MR simulation.

MR simulation was performed on a 3T clinical MRI scanner (MAGNETOM Skyra, Siemens Healthineers, Forchheim, Germany) located within the Department of Radiology using a 15‐channel head coil specifically designed to accommodate radiotherapy immobilization devices (Encompass, CQ Medical, Avondale, PA, USA).^11^ An 11‐min MR simulation protocol for synthetic CT generation was appended to the standard diagnostic MRI protocol (25–30 min).

The sCT protocol consisted of four MRI sequences: T1 VIBE Dixon, T2 SPACE, PETRA, and TOF‐MRA.^4^, ^12^ MRI datasets were subsequently imported into a workstation (syngo.via, Siemens Healthineers, Forchheim, Germany) for sCT generation.

The pCT and MRI datasets were imported into the clinical treatment planning system (Varian Eclipse). Clinical treatment plans were generated using the pCT datasets. Post‐contrast T1 VIBE images acquired as part of the diagnostic MRI protocol were registered to the pCT for target and OAR delineation. Subsequently, secondary research treatment plans were recalculated on the sCT datasets using identical planning parameters.

## 3 RESULTS

Twenty‐eight patients underwent brain MR simulation. Twenty‐five synthetic CT datasets were successfully generated and retrospectively reviewed.

### 3.1 Case exclusion

Three patients were excluded from analysis (approximately 11% of 28 cases): one because of compromised sCT quality caused by coil connection failure, one because of local data loss before transfer to the syngo.via processing workstation, and one because the patient withdrew from the clinical trial.

### 3.2 Workflow‐related sCT challenges

Among the 25 successfully generated sCT datasets, findings were categorized into two groups: workflow‐related sCT challenges and algorithm‐related imaging artifacts.

Workflow‐related challenges included Inter‐sequence patient motion (9/25 cases, 36%), external localization marker interference (10/25 cases, 40%) and suboptimal coil placement (1/25 cases, 4%)

Algorithm‐related imaging artifacts included HU misclassification at tissue/bone/air interfaces (16/25 cases, 64%) such as Bone HU underestimation and Air cavity filling, and sCT degradation associated with post‐operative anatomy and surgical meshes (2/25 cases, 8%)

#### 3.2.1 Inter‐sequence patient motion

In nine cases (36% of 25 cases), spatial misalignment between post‐contrast VIBE images and the generated sCT datasets was observed and was likely attributable to inter‐sequence patient motion. Rigid re‐registration was performed when rotational misalignment exceeded 1° during image review.

#### 3.2.2 External localization markers

**FIGURE 2:**
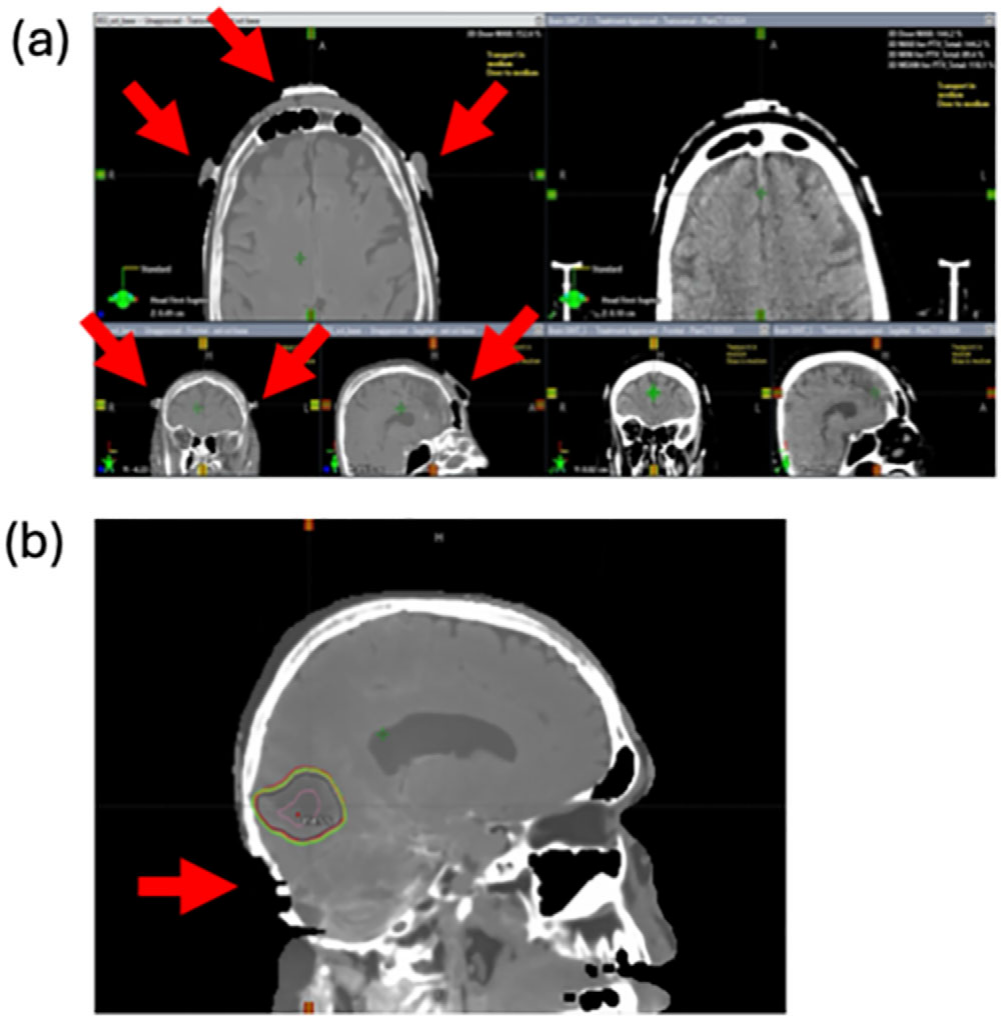
(a) Comparison between sCT (left) and pCT (right) focused on the bone/air interface near the sinus (b) sCT artifacts shown near the cerebellum due to the coil misplacement. Parts of skin and skull were classified as air.

The presence of external materials within the imaging field of view affected sCT generation despite clinically acceptable MRI image quality (10/25 cases, 40%). Examples included lipid‐filled MR localization markers attached to the immobilization mask (Figure 2a). Signal perturbation associated with MR localization markers was observed in all evaluated cases in which the markers were used (10/10 cases, 100%).

#### 3.2.3 Suboptimal coil placement

Figure 2b demonstrates artifacts arising from suboptimal posterior receiver coil positioning. Inadequate coil coverage resulted in signal non‐uniformity within the inferior‐posterior skull, which propagated into the generated sCT. Portions of the skin and skull were subsequently classified as air. This finding occurred in 1 of 25 cases (4%). The MRI images appeared clinically acceptable; however, the resulting sCT quality was substantially degraded.

### 3.3 Imaging artifacts (algorithm‐related)

#### 3.3.1 HU misclassification at tissue/bone/air interfaces

HU misclassification at tissue/bone/air interfaces represented the most common imaging artifact in this cohort, occurring in 16 of 25 cases (64%). Two subtypes were identified: air cavity filling and bone HU underestimation.

Air cavity filling occurred when air‐containing regions near the sinus or skull base were incorrectly classified as bone. Bone HU underestimation occurred when skull bone attenuation values were systematically underestimated. Both findings likely arose from the inherent difficulty of differentiating low‐signal structures such as cortical bone and air using MRI signal intensity alone. These inaccuracies produced dosimetric deviations relative to pCT‐based dose calculations.

**FIGURE 3:**
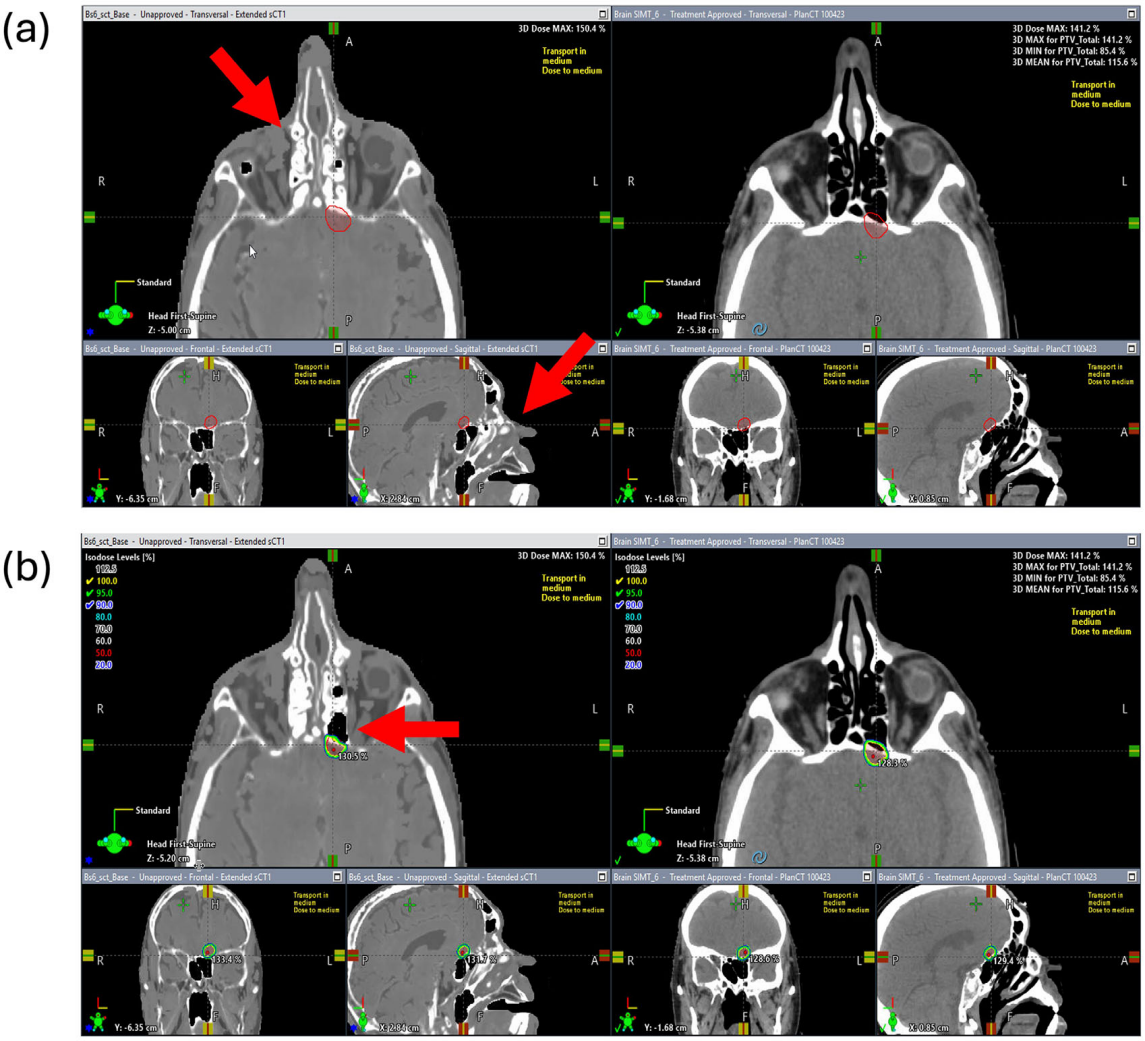
Comparison between sCT (left) and pCT (right) focused on the bone/air interface near the sinus (a) Example images where the HU misclassification was noticeable (b) Example images where the dose discrepancy was noticeable.

Figure 3a illustrates an example of HU misclassification near the sinus where air cavities were incorrectly classified as bone, resulting in air cavity filling artifacts. The resulting isodose lines were displaced relative to those from the planning CT, producing reduced target coverage (Figure 3b).

**FIGURE 4:**
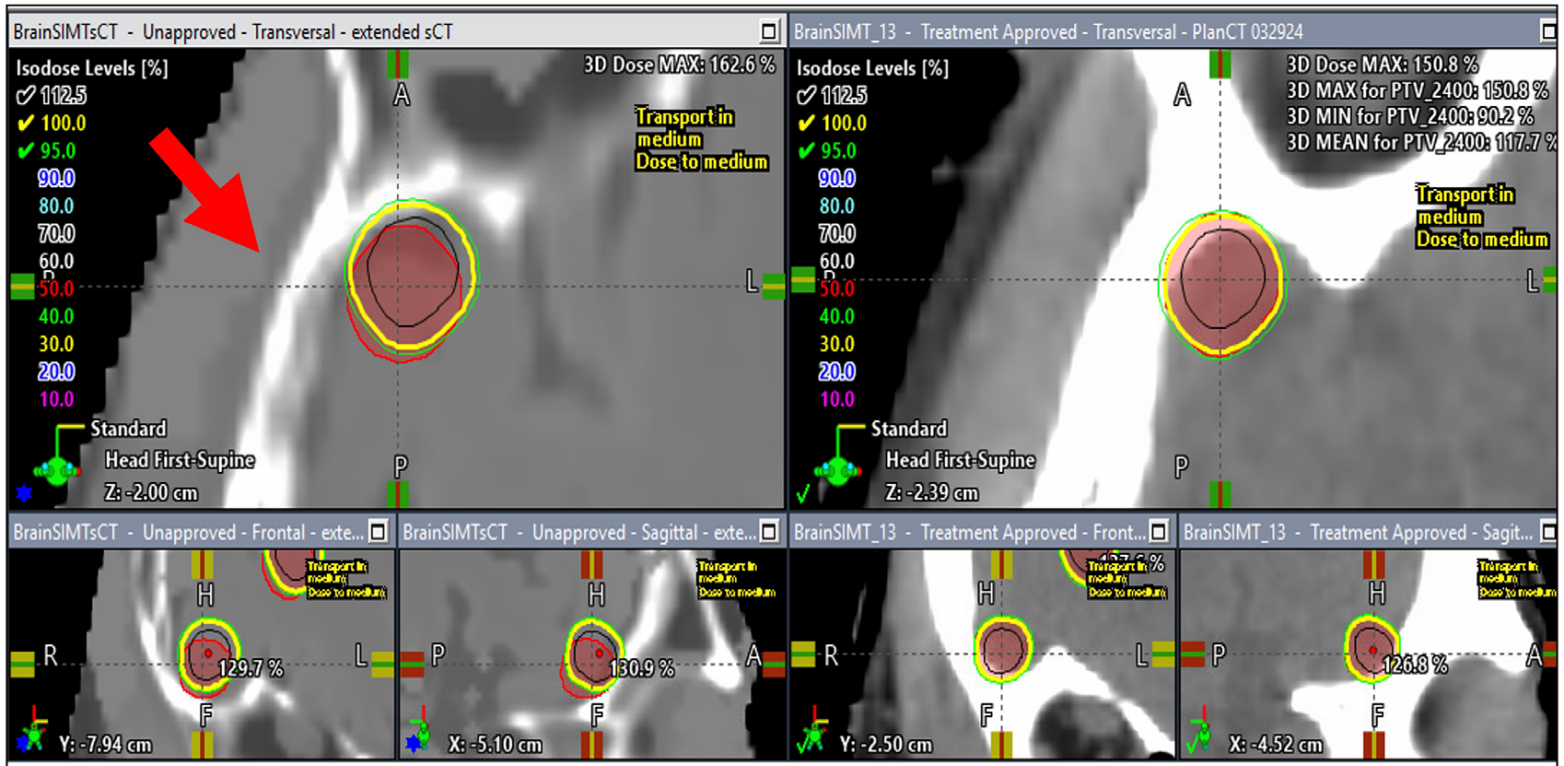
Comparison between sCT (left) and pCT (right) focused on the bone/tissue interface in the right frontal lobe. The targets are shown in red.

Figure 4 demonstrates bone HU underestimation in the frontal skull adjacent to a treatment target. Dose distributions calculated on the sCT differed from those on the pCT, again resulting in reduced target coverage.

#### 3.3.2 Post‐operative anatomy and surgical meshes

**FIGURE 5:**
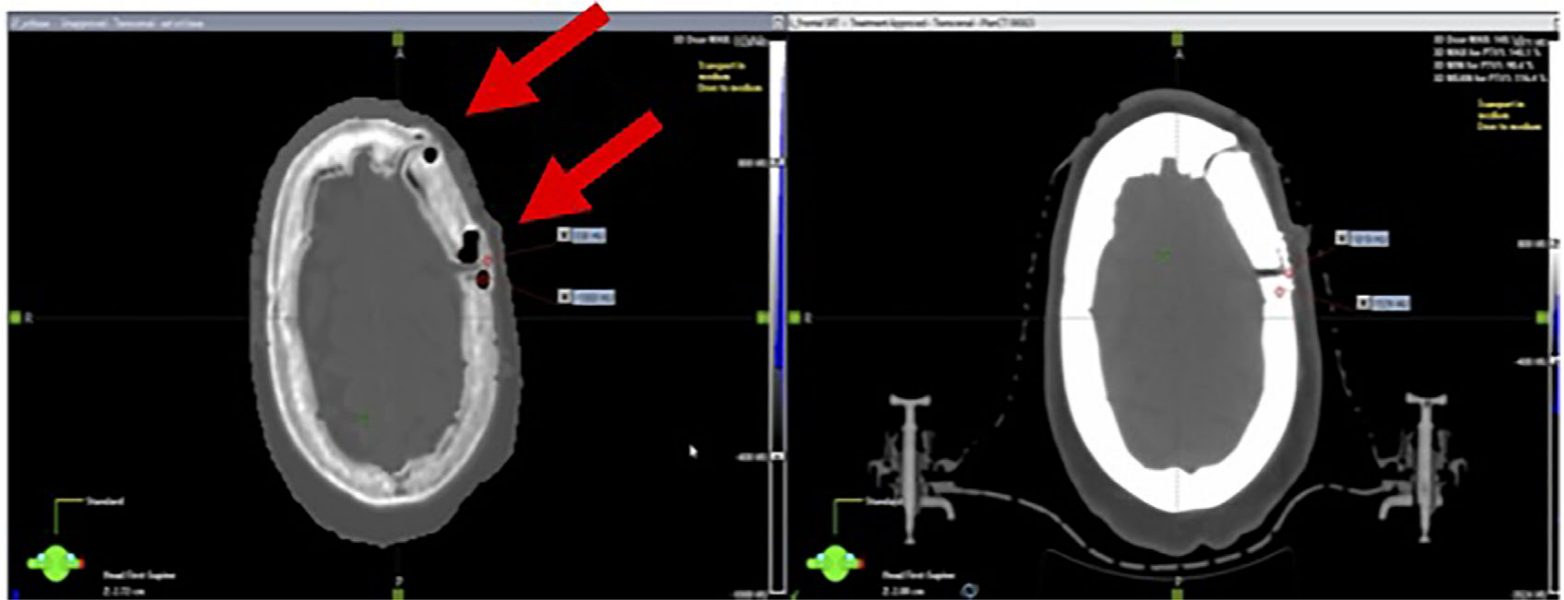
Comparison between sCT and CT images where the anatomy of the patient was altered and surgical meshes were present.

In 2 of 25 cases (8%), the presence of surgical meshes near post‐operative sites resulted in substantial sCT misclassification. Figure 5 shows Bone adjacent to the surgical site was classified as air, while the meshes themselves were classified as bone. Of the 25 cases, 5 patients presented with post‐operative anatomy. In 2 of these 5 cases (40%), sCT signal perturbation was observed in the region of surgery.

This finding reflects an inherent limitation of atlas‐based algorithms, which rely on anatomical atlases derived from normal anatomy. Performance in patients with substantially altered post‐operative anatomy has not been fully validated. Unlike workflow‐related challenges, this issue cannot be resolved through procedural modifications alone and therefore represents an important patient selection consideration.

### 3.4 Supplementary materials: Commissioning and per‐patient QA checklists

Based on the implementation experience described above, two structured checklists were developed to support safe clinical adoption of the brain MR‐only workflow.

Supplementary Material 1 provides a commissioning checklist addressing MR scanner logistics and workflow integration, Coil selection and qualification, sCT algorithm verification, Ongoing QA procedures, Re‐verification following software or hardware changes, End‐to‐end testing before clinical implementation.

Supplementary Material 2 provides a per‐patient QA checklist addressing Patient eligibility, Pre‐scan preparation, Real‐time monitoring during acquisition, and Post‐scan physics review and sCT approval.

Together, these checklists translate the implementation challenges identified in this study into actionable quality assurance procedures for institutions implementing MR‐only brain radiotherapy workflows.

## 4 DISCUSSION

CT simulation became a standard component of radiation oncology workflows with the development of three‐dimensional conformal radiotherapy and dedicated radiotherapy CT scanners.^13^ MR simulation is currently undergoing similar development and may become practice‐changing by enabling MR‐only simulation and treatment workflows. This work should be interpreted as a clinical implementation experience emphasizing practical limitations and QA considerations. Findings identified in this study were divided into workflow‐related challenges and algorithm‐related imaging artifacts.

Our implementation utilized a diagnostic radiology MRI scanner rather than a dedicated MR simulator within the radiation oncology department. Consequently, additional coordination between radiation oncology and radiology personnel was required. For example, a radiation therapist was required to be present during MR simulation to ensure appropriate immobilization and positioning consistent with treatment delivery.

The absence of a dedicated MR simulator also limited our ability to standardize MR acquisition protocols and minimize inter‐sequence delays. This scenario likely reflects the experience of many institutions attempting to implement MR‐based or MR‐only workflows without dedicated MR simulation infrastructure.

Workflow‐related challenges—including inter‐sequence motion, suboptimal coil placement, and external localization marker interference—arose from procedural or logistical factors and may potentially be mitigated through workflow optimization.

Nine of the 25 evaluated cases required rigid re‐registration because of inter‐sequence patient motion. This misalignment was likely attributable to patient motion occurring between MRI sequences. In our institutional workflow, target delineation is performed using post‐contrast T1 VIBE images rather than the T1 VIBE sequence used for sCT generation. Because the post‐contrast sequence is acquired approximately 20–25 min after the sCT acquisition sequences, temporal separation increases the likelihood of inter‐sequence motion. This is particularly relevant in cases where additional sequences were acquired beyond the standard sCT protocol, further prolonging total scan time.

Placement of external localization markers is a standard component of CT‐based simulation workflows. Because conventional metallic BB markers cannot be used during MRI acquisition, lipid‐filled MR‐compatible markers were used instead for 10 cases. However, because these markers were not represented within the anatomical atlas used by the sCT algorithm, they occasionally resulted in tissue misclassification.

To mitigate this effect, the use of MR‐compatible external localization markers should be carefully evaluated during MR‐only workflow implementation. During treatment planning, external markers may be overridden as air in the pCT because metallic BB markers and/or wires, when present, can be overwritten. When feasible, anatomical landmarks may serve as alternative localization references.

MR coil placement introduced an additional implementation challenge. Proper receiver coil positioning is critical for maintaining adequate signal quality and uniformity.^14^, ^15^, ^16^ However, coil placement remains operator dependent, and suboptimal positioning may occur. In such situations, MRI datasets may remain diagnostically acceptable while the corresponding sCT quality is degraded.

In contrast, algorithm‐related imaging artifacts—including HU misclassification at tissue/bone/air interfaces and degradation associated with post‐operative anatomy—reflect fundamental limitations of atlas‐based tissue classification.

HU misassignment at tissue/bone/air interfaces may significantly affect dose calculation accuracy. Because both bone and air exhibit low MRI signal intensity, differentiation between these tissues remains challenging. Air may therefore be incorrectly classified as bone, resulting in air cavity filling artifacts, while skull HU values may also be underestimated. For VMAT treatment plans, attenuation varies according to gantry angle and beam path through heterogeneous tissues. Consequently, HU inaccuracies may produce anisotropic dose perturbations and reduce target dose conformality.

Tissue misclassification may occur in the presence of metallic foreign bodies such as surgical clips and meshes frequently encountered in post‐operative brain radiotherapy. Although metal artifacts on CT imaging are well recognized, their manifestation on sCT images differs substantially.

Because the performance of the atlas‐based algorithm in abnormal anatomy has not been fully validated, the resulting sCT datasets may become misleading in post‐operative or anatomically altered patients. In our cohort, surgical meshes resulted in adjacent bone being classified as air while the meshes themselves were classified as bone.

Another important implementation consideration concerns long‐term maintenance of sCT quality following system modifications. MRI hardware degradation, software upgrades, sequence modifications, coil replacement, and algorithm updates may alter MRI signal characteristics sufficiently to affect sCT generation performance.

Because the relationship between MRI signal intensity and sCT HU assignment is closely linked to the system configuration used during commissioning, such changes may invalidate commissioning assumptions. Institutions implementing MR‐only workflows should therefore establish structured re‐verification procedures following hardware or software modifications.

These procedures should include evaluation of sCT HU comparison, Geometric fidelity, Representative dosimetric verification. This recommendation is incorporated into the commissioning checklist provided in Supplementary Material 1.

To address the per‐patient challenges identified in this cohort—including inter‐sequence motion, coil placement, external markers, and post‐operative anatomy—Supplementary Material 2 was developed as a structured per‐patient QA checklist to be completed at each stage of the clinical workflow.

This study has limitations. The workflow utilized an atlas‐based algorithm before the vendor's deep learning–based sCT reconstruction method became available. Although the atlas‐based workflow was maintained for methodological consistency, atlas‐based algorithms are known to be sensitive to discrepancies between atlas anatomy and patient anatomy.^17^

The atlas‐based algorithm used in this study functions fundamentally as a tissue classifier that assigns probabilities for gray matter, white matter, fat, bone, and CSF/blood based on MRI signal intensity and anatomical atlas location. Because tissue classification relies on multiple MRI sequences, the approach is vulnerable to inter‐sequence motion.

In contrast, the newer deep learning–based algorithm generates sCT images from a single T1 VIBE Dixon sequence and therefore may reduce vulnerability to inter‐sequence patient motion. The deep learning–based approach utilizes a densely connected UNet combined with a conditional generative adversarial network (GAN) to generate sCT images directly from a single MRI sequence. Although our study utilized an atlas‐based method, many of the observed implementation challenges are workflow‐ and anatomy‐dependent and may therefore persist regardless of the underlying reconstruction algorithm. Institutions transitioning to the deep learning–based method should therefore maintain the workflow safeguards described in this report.

## 5 CONCLUSION

This study describes clinical implementation experience with a brain MR‐only radiotherapy workflow using an atlas‐based synthetic CT algorithm, with emphasis on practical implementation challenges rather than systematic dosimetric validation.

Among 28 enrolled patients, 25 sCT datasets were successfully generated. Findings were classified into workflow‐related sCT challenges—including inter‐sequence patient motion, suboptimal coil placement, and external localization marker interference —and algorithm‐related imaging artifacts, including HU misclassification at tissue/bone/air interfaces and sCT degradation associated with post‐operative anatomy and surgical meshes. This distinction has important practical implications. Workflow‐related challenges may be mitigated through procedural modification and quality assurance processes, whereas algorithm‐related artifacts require careful case‐specific review and, in some cases, patient selection criteria.

These findings demonstrate that real‐world MR‐only implementation introduces complexities not fully represented in controlled validation studies. Case‐specific review of each synthetic CT by a qualified medical physicist, supported by structured eligibility screening and standardized per‐patient QA procedures, is essential before clinical implementation. Furthermore, prospective site commissioning—including sCT algorithm verification, coil qualification, and structured re‐verification procedures following system modifications—is critical for maintaining long‐term workflow integrity. The commissioning and per‐patient QA checklists provided as supplementary materials offer a practical framework for institutions implementing or expanding MR‐only brain radiotherapy workflows.

## AUTHOR CONTRIBUTIONS

Hyeri Lee, Ahmet S Ayan, Sasha Beyer, Dukagjin Blakaj, Dominic DiCostanzo, John C Grecula, Joshua Palmer, Raju Raval, Raj Singh, Evan Thomas, Michael Weldon, Xiangyu Yang, Wesley Zoller and Nilendu Gupta contributed to the conception and design of the study and contributed to data acquisition. Hyeri Lee analyzed and interpreted all data and wrote the draft. All authors reviewed and revised the final manuscript.

## CONFLICT OF INTEREST STATEMENT

The authors declare no conflicts of interest.

## ETHICS STATEMENT

This study was approved by the institutional ethics committee.

## CONSENT STATEMENT

The authors consent to the publication of this manuscript.

## Supporting information



Supporting Information

Supporting Information
