# Supplementary material for: Synthetic CT artifacts in MR‐only brain radiotherapy: Clinical implementation experience
Source: J Appl Clin Med Phys. 2026 Aug 3;27(8):e70733. doi: 10.1002/acm2.70733 (PMC13433486; doi:10.1002/acm2.70733)
Supplement: Supplementary file 1 [file ACM2-27-e70733-s001.docx]

**Commissioning Checklist: Brain MR-only Radiotherapy Workflow**

*Site-readiness and commissioning tasks to be completed before the MR-only workflow is opened for clinical use. All sections should be completed in order. Outstanding items must be resolved and signed off before proceeding to the end-to-end test.*

# 1. MR Scanner / Simulator Location

*The physical location of the MR scanner determines staffing, logistics, and image transfer requirements. Complete this section for your specific site configuration.*

| **Item** | **Details / Considerations** | **Location / Context** | **Action Required** | **Responsible Person** |
| --- | --- | --- | --- | --- |
| Scanner in RadOnc | MR simulator physically located within the Radiation Oncology department | RadOnc | Confirm mask-making facility is available on-site or nearby; establish internal transport protocol if needed; confirm RTT availability for scanning | Physicist / RTT Lead |
| Scanner in Radiology | MR scanner located in Radiology department; workflow crosses departmental boundaries | Radiology | Agree inter-departmental protocol for patient scheduling, mask transport, and image transfer; confirm whether Radiology MR Tech or RadOnc RTT will operate scanner | Physicist / RadOnc & Radiology Leads |
| Scanner at other location | MR scanner at external or off-site location (e.g. separate building, affiliated hospital) | External | Establish formal patient transport and image transfer pathway; define responsibilities for each site; agree on contingency if scan cannot proceed | Physicist / Department Manager |
| Mask fabrication — location and timing | Where will the immobilization mask be made, and at what point in the workflow? | All locations | Define and document mask-making site and timing relative to MR appointment; ensure equipment and trained staff are available at that location | RTT Lead |
| Mask transport (if applicable) | Does the mask need to be moved between fabrication site and MR scanner location? | Multi-site workflows | Establish transport procedure; confirm mask integrity check on arrival; document responsibility for transport | RTT Lead |
| Personnel requirements | Does the MR simulation workflow require additional staff beyond standard RT staffing (e.g. RTT, MR Tech)? | All locations | Identify staffing gaps; agree roles and responsibilities for each professional group; confirm MR safety training requirements for all staff entering MR suite | Department Manager / Physicist |
| Image transfer to planning software | How will MR and synthetic CT (sCT) images be transferred from MR scanner to the RT planning system? | All locations | Confirm DICOM transfer pathway (direct DICOM push, PACS intermediary, manual export); test end-to-end transfer before clinical use; document and validate transfer protocol | Physicist / IT |

# 2. MRI Coil Types

*All coils intended for clinical use must be verified for compatibility with the sCT algorithm and assessed for acceptable image quality before commissioning is complete.*

| **Item** | **Details / Considerations** | **Location / Context** | **Action Required** | **Responsible Person** |
| --- | --- | --- | --- | --- |
| Vendor-provided (OEM) coils | Coils supplied and validated by the MR scanner manufacturer as part of the standard system | MR suite | Confirm coil is listed as supported for sCT acquisition in vendor documentation; include in routine coil QA program | Physicist / MR tech |
| Specialty coils (non-OEM) | Coils not supplied by the MR scanner manufacturer (e.g. RT-specific flex coils, third-party coils) | MR suite | Check with MR simulator manufacturer whether the coil is supported for sCT generation; obtain written confirmation if possible | Physicist |
| sCT image quality — coil acceptance | Is the MRI and resulting sCT quality clinically acceptable when using the intended coil(s)? | All coil types | Acquire test images with each coil configuration; review MRI and sCT quality with Radiation Oncologist and/or Radiologist; document acceptance decision before clinical use | Physicist / Radiation Oncologist / Radiologist |

# 3. sCT Algorithm Commissioning

## 3a. Algorithm Investigation

*Gather sufficient information about the algorithm to inform a safe and appropriate verification strategy.*

| **Item** | **Details / Considerations** | **Location / Context** | **Action Required** | **Responsible Person** |
| --- | --- | --- | --- | --- |
| Vendor documentation review | Review manufacturer's technical documentation for the sCT algorithm | Pre-commissioning | Identify supported anatomical sites, patient populations, and known limitations; document findings | Physicist |
| Literature review | Review published peer-reviewed evidence for the specific sCT algorithm in clinical use | Pre-commissioning | Assess reported accuracy, failure modes, and dosimetric impact; identify any site-specific considerations relevant to your patient population | Physicist |
| Supported populations and anatomy | Confirm which patient populations and anatomical regions are validated for the algorithm | Pre-commissioning | Document any exclusions (e.g. pediatric, post-operative, implants); align with eligibility criteria in per-patient QA checklist | Physicist |
| Scanner / software / coil compatibility | Confirm algorithm compatibility with your specific MR scanner model, software version, and intended coil types | Pre-commissioning | Obtain written confirmation from vendor; document scanner software version at time of commissioning; flag need to re-verify after software updates | Physicist |
| Regulatory / clinical release status | Confirm the algorithm holds appropriate regulatory clearance (e.g. CE mark, FDA clearance) for clinical use at your site | Pre-commissioning | Obtain documentation of clearance; confirm intended use aligns with cleared indication; keep on file | Physicist / Department Manager |

## 3b. Algorithm Verification

*Demonstrate that the algorithm performs to a clinically acceptable standard at your site before use in patients.*

| **Item** | **Details / Considerations** | **Location / Context** | **Action Required** | **Responsible Person** |
| --- | --- | --- | --- | --- |
| Geometric fidelity | Assess dimensional accuracy of sCT relative to reference CT | Verification cohort | Measure key anatomical dimensions on sCT and reference CT; confirm geometric fidelity is within clinically acceptable tolerance; document results | Physicist |
| CT comparison — patient cohort | Compare sCT with reference CT across a representative cohort of patients (sufficient to cover expected anatomical variation) | Verification cohort | Assess HU accuracy, tissue classification, and structural integrity across cohort; document any systematic deviations or failure cases | Physicist |
| Dosimetric validation | Validate that dose calculated on sCT is equivalent to dose calculated on reference CT | Verification cohort | Perform dose calculation on both sCT and CT for each patient; compare using gamma analysis and/or DVH metrics; document pass rates and any clinically significant deviations | Physicist |
| Clinical sign-off | Formal acceptance of the sCT algorithm for clinical use | Post-verification | Physicist and Radiation Oncologist to review verification results; document acceptance with signatures and date; define tolerance thresholds for ongoing use | Physicist / Radiation Oncologist |

# 4. Ongoing QA Program

*Establish QA procedures before go-live. The program must cover both routine periodic QA and re-verification triggered by hardware or software changes.*

## 4a. Routine Periodic QA

*Define test, frequency, tolerance, and responsibility for each QA item before clinical go-live.*

| **Item** | **Details / Considerations** | **Location / Context** | **Action Required** | **Responsible Person** |
| --- | --- | --- | --- | --- |
| Geometric accuracy — daily | Confirm MR geometric accuracy is within tolerance prior to clinical scanning | Daily | Define daily geometric QA test and pass/fail criteria; document in QA log; establish action levels | MR Tech / Physicist |
| Geometric accuracy — monthly/annual | More comprehensive geometric accuracy assessment across the imaging volume | Monthly / Annual | Define test protocol and tolerances; assign responsibility; document results | Physicist |
| HU consistency | Monitor sCT HU values over time to detect algorithm drift or scanner performance changes | Monthly / Annual | Define reference HU values and acceptable tolerance bands from commissioning data; perform periodic checks; trend over time | Physicist |
| Coil functionality | Verify all coil elements are functioning correctly and SNR is within acceptable range | Daily / Monthly | Define coil QA test (SNR measurement or vendor-provided coil check); establish pass/fail criteria; document results | MR Tech / Physicist |

## 4b. QA Following Hardware / Software Updates

*Any change to the MR system, software, algorithm, or coils must trigger appropriate re-verification before the workflow returns to clinical use.*

| **Item** | **Details / Considerations** | **Location / Context** | **Action Required** | **Responsible Person** |
| --- | --- | --- | --- | --- |
| MR scanner hardware update | Any hardware change to the MR scanner (e.g. gradient coil, RF system replacement) | As required | Repeat relevant components of commissioning verification; document re-verification before returning to clinical use | Physicist |
| MR sequence update | Change to the sCT acquisition sequence parameters | As required | Re-verify sCT image quality and dosimetric accuracy after sequence change; update commissioning records | Physicist |
| sCT algorithm update | New version of the sCT algorithm software | As required | Treat as partial re-commissioning; repeat dosimetric validation on a representative patient cohort; obtain clinical sign-off before returning to use | Physicist / Radiation Oncologist |
| MR scanner software version update | System software upgrade (not specific to sCT algorithm) | As required | Verify sCT output is unchanged post-update; check coil compatibility; document version change and re-verification outcome | Physicist |
| Coil replacement | Replacement of an existing clinical coil with a new unit (same model) | As required | Perform coil QA and sCT image quality check with new coil before clinical use; confirm replacement unit performs equivalently to the original | Physicist / MR Tech |

# 5. End-to-End Test

*The end-to-end test must be completed successfully before the first clinical patient is treated. It validates the entire workflow from immobilisation through to plan delivery readiness.*

| **Item** | **Details / Considerations** | **Location / Context** | **Action Required** | **Responsible Person** |
| --- | --- | --- | --- | --- |
| Patient simulation | Simulate a representative patient (or anthropomorphic phantom) through the complete MR-only workflow from immobilization to planning | Pre-clinical go-live | Complete all steps: mask fabrication, MR acquisition, sCT generation, image transfer, registration, dose calculation; document each step | Physicist / RTT |
| Image transfer verification | Confirm all image sets (MRI, sCT) transfer correctly and completely to the planning system with correct patient demographics and DICOM tags | Pre-clinical go-live | Check DICOM metadata, image orientation, and completeness at planning system; confirm no transfer errors | Physicist / IT |
| Registration and contouring | Confirm sCT can be correctly registered to planning MRI and that contours can be drawn | Pre-clinical go-live | Perform registration in planning system; assess geometric accuracy; confirm contouring tools function correctly on sCT | Physicist / Radiation Oncologist |
| Dose calculation | Confirm dose calculation on sCT is feasible and produces clinically plausible results | Pre-clinical go-live | Calculate a representative treatment plan on sCT; compare DVH metrics and dose distribution with CT-based reference if available | Physicist |
| Plan export and delivery readiness | Confirm treatment plan can be exported from TPS and is deliverable on the treatment unit | Pre-clinical go-live | Export plan; verify on record-and-verify system; confirm no errors in machine parameters or patient setup data | Physicist / RTT |
| End-to-end sign-off | Formal acceptance that the complete workflow has been tested and is ready for clinical use | Pre-clinical go-live | Document end-to-end test results; obtain signatures from Physicist and Radiation Oncologist; set go-live date | Physicist / Radiation Oncologist / Department Manager |
