# Supplementary material for: Synthetic CT artifacts in MR‐only brain radiotherapy: Clinical implementation experience
Source: J Appl Clin Med Phys. 2026 Aug 3;27(8):e70733. doi: 10.1002/acm2.70733 (PMC13433486; doi:10.1002/acm2.70733)
Supplement: Supplementary file 2 [file ACM2-27-e70733-s002.docx]

**QA Checklist: Brain MR-only Radiotherapy Workflow**

*Covers patient eligibility through to post-scan physics sign-off. All sections must be completed in order. Any exclusion or flag not resolved before the next stage requires escalation to the responsible Physicist.*

# 1. Eligibility

*Complete before booking the MR scan. Patients failing any exclusion criterion should be referred to the CT-based workflow.*

| **QA Item** | **What to Check** | **Potential Issues** | **Recommended Action** | **Responsible Person** |
| --- | --- | --- | --- | --- |
| Patient Age | Is the patient pediatrics? | Synthetic CT (sCT) algorithm may not have been validated for pediatrics population | Exclusion — refer to CT-based workflow | Physicist / Therapist |
| Claustrophobia / Anxiety | Does the patient have known claustrophobia or significant anxiety? | Patient may be unable to tolerate MR scan; motion artifacts if anxious | Discuss with clinical team; consider anxiolytic pre-medication or exclusion | Therapist / Physician |
| Acute Renal Failure | Does the patient have acute renal failure? | Gadolinium contrast is contraindicated in renal failure; contrast scan should not proceed | Check if physician has requested contrast; if so, flag to clinical team before proceeding | Therapist / Physician |
| MR-Safe Implants | Does the patient have non-MR-compatible implants? | Unable to perform MR scan due to safety risk | Exclusion — refer to CT-based workflow | Therapist |
| Implant Location (MR-safe) | If implant is MR-safe, is it within or near the scan FOV? | Metallic implants within or adjacent to FOV may degrade sCT quality even if MR-safe | Flag for enhanced post-scan sCT review; consider CT-based workflow if in critical region | Physicist / Therapist |

# 2. Pre-scan

*Complete on the day of scanning, before the patient enters the MR suite.*

| **QA Item** | **What to Check** | **Potential Issues** | **Recommended Action** | **Responsible Person** |
| --- | --- | --- | --- | --- |
| Patient Selection | Post-operative anatomy (implant, abnormal OAR anatomy) | Algorithm may fail in non-standard anatomy | Flag for enhanced post-scan review or consider CT-based workflow | Physicist |
| Landmark / Isocenter Positioning | Correct positioning to ensure adequate skull base and vertex coverage within sCT FOV | Inadequate coverage leads to incomplete sCT; dosimetric errors at field edges | Reposition before scan; confirm coverage on scout | Therapist |
| Immobilization Mask — Availability | Confirm mask is available and correctly labelled | — | Make or locate mask before patient arrives | Therapist |
| Immobilization Mask — Stability | Check mask fit and patient comfort | Motion artifacts if mask poorly fitted or patient uncomfortable | Adjust fit; ensure patient comfort before proceeding | Therapist |
| MR Protocol Verification | Confirm correct sCT protocol loaded; correct FOV, orientation, and sequence order | Wrong protocol leads to incorrect sCT generation or missing sequences | Verify on scanner console before patient enters bore; correct if needed | Therapist / Physicist |
| Coil Connection and Functionality | Correct connection of all elements; no inactive channels; system coil check passed | Reduced SNR, regional signal loss, non-uniform intensity — all compromise sCT quality | If unacceptable, do not proceed; consider CT-based workflow | Therapist |
| Coil Placement | Correct placement and coverage over region of interest | Inadequate coverage leads to signal dropout and sCT artefacts | Reposition coil before scan | Therapist |

# 3. During Scan

*Therapist to monitor and document in real time during the MR acquisition.*

| **QA Item** | **What to Check** | **Potential Issues** | **Recommended Action** | **Responsible Person** |
| --- | --- | --- | --- | --- |
| Inter-sequence Movement | Can patient tolerate scan without interruption between sequences? | Inter-sequence motion degrades sCT quality and causes misregistration | Flag for artifacts and registration review post-scan | Therapist |
| Intra-sequence Motion (sCT) | Monitor patient for motion during the sCT sequence specifically | The sCT sequence is particularly sensitive to intra-sequence motion; can produce severe sCT artifacts | Pause and repeat the sequence if motion observed; document | Therapist |
| Additional MR Sequences | Were additional sequences acquired beyond the standard sCT protocol? | Lengthy scan time increases motion risk and misregistration probability | Document any additional sequences; flag for potential registration impact at physics review | Therapist |
| Total Scan Time | Record actual total scan time | — | Document in patient record for audit and QA purposes | Therapist |

# 4. Post-scan Physics Review

*Physicist to complete before the sCT is released for treatment planning. sCT must not proceed to planning without formal sign-off.*

| **QA Item** | **What to Check** | **Potential Issues** | **Recommended Action** | **Responsible Person** |
| --- | --- | --- | --- | --- |
| Review Checklist Items 1–3 | Review all flags and warnings raised during eligibility, pre-scan, and during-scan stages; Data transfer to treatment planning system | Unresolved flags may indicate compromised image quality or patient safety concerns | Incorporate all flags into image quality review before accepting sCT | Physicist |
| sCT Quality — HU/ED Plausibility | Are sCT HU values within expected ranges for brain tissue, bone, and air cavities? | Out-of-range values indicate algorithm failure and will cause dosimetric errors | Compare against reference sCT; reject and revert to CT-based workflow if out of range | Physicist |
| sCT Quality — Visual Integrity | Skull integrity; air cavities; tissue classification; external material regions; overall scan quality | Compromised sCT will impact dosimetry | Determine if sCT is acceptable; document outcome; escalate if uncertain | Physicist |
| Geometric Distortion | Assess for MR geometric distortion artefacts, particularly at skull base | MR distortion near skull base can affect dosimetry in critical OAR regions | Flag significant distortion; consider CT-based workflow if dosimetrically relevant | Physicist |
| Registration Quality | Assess quality of sCT-to-planning MRI registration | Poor registration leads to systematic dosimetric error throughout the plan | Review overlay carefully; re-register or reject sCT if registration inadequate | Physicist |
| sCT Acceptance Sign-off | Has the sCT been reviewed and formally accepted by a Physicist? | Unsigned-off sCT must not proceed to planning | Document acceptance (or rejection) with physicist name and date; if rejected, initiate CT-based workflow | Physicist |
